# Editorial: Epigenetics in the microbiome-host crosstalk: from mechanisms to therapeutics

**DOI:** 10.3389/fimmu.2025.1589656

**Published:** 2025-03-19

**Authors:** Omar Ramos-Lopez, Chengfei Liu, Hao-Yu Liu

**Affiliations:** ^1^ Medicine and Psychology School, Autonomous University of Baja California, Tijuana, Baja California, Mexico; ^2^ Department of Urologic Surgery, University of California, Davis, Davis, CA, United States; ^3^ Davis Comprehensive Cancer Center, University of California, Davis, Davis, CA, United States; ^4^ College of Animal Science and Technology, Yangzhou University, Yangzhou, China; ^5^ Joint International Research Laboratory of Agricultural & Agri-Product Safety, The Ministry of Education of China, Yangzhou University, Yangzhou, China

**Keywords:** dysbiosis, epigenetic, bacteria, microbiota, microbiome, therapeutics

In recent years, the study of the gut microbiota has gained significant interest due to its implications on the health status of the host and its capacity to influence host responses to environmental cues. The human digestive system harbors trillions of microorganisms that contribute to important functions in the host, including nutrient metabolism, intestinal permeability, and immune responses. The molecular mechanisms underlying these biological processes include epigenetic phenomena that may alter gene expression without modifying the DNA sequence, ultimately influencing the cell phenotype and the host physiology (1). Notably, the intestinal microbiota participates in the biosynthesis of substrates for DNA/histone methylation reactions as well as the regulation of epigenetically active enzymes and activation of cellular processes involving epigenetic pathways. Of note, emerging research has linked perturbation of these microbiota-epigenetic interactions with the onset and progression of immunological disorders, metabolic syndrome features, cancer, and neurological pathologies (2). This Research Topic includes nine original articles and one review that explore the gut microbiota-epigenetics connections involved in disease physiopathology, with potential implications for novel therapeutic interventions.

To analyze global research trends regarding intestinal microbiota and epigenetics, Tian and Chen performed a comprehensive bibliometric analysis to visualize the body of knowledge and research priorities in this field. They found that gut microbiota and epigenetics are closely related to pathologies such as breast and colorectal cancer, inflammatory bowel disease, and psychiatric disorders. Additionally, they underscored the potential of diet (probiotics) and a healthy lifestyle to regulate gut microbiota and reduce the burden of these diseases. Using a related bibliometric approach, Tang et al. systematically analyzed *Cryptococcus* species and their dynamism with the host immune system. They found that current research reveals intricate interactions between *Cryptococcus* pathogenesis (mechanisms and complications) and host immunity, with implications in the development of immunotherapies and visualizing critical directions in this domain. Furthermore, Shi et al. identified *DRAM1*, *PSTPIP2*, and *UPP1* as differentially expressed genes in *Staphylococcus aureus* bloodstream infection using human and mouse samples and an integrative bioinformatics analysis, highlighting their potential as diagnostic biomarkers of this infection.

Mendelian randomization is increasingly used in human biology to assess the causal effects of modifiable risk factors on health outcomes by using genetic information from the host. In this Research Topic, Zhu et al. explored the causal association between microbiota and skin appendage disorders using Mendelian randomization. They found relevant causal relationships between genetic liability in the skin and gut microbiota with skin appendage disorders. Specifically, they identified several skin bacteria (*Staphylococcus, Streptococcus, and Propionibacterium*) as being positively associated, and *Bifidobacteria and Lactobacilli* as probiotics exerting a protective effect on this disorder. Similarly, Zhang et al. identified causal relationships between gut microbiota and premature rupture of membranes using a Mendelian randomization analysis. The results revealed that class *Mollicutes*, genus *Marvinbryantia*, genus *Ruminooccaceae UCG003* and phylum *Tenericutes* were associated with a reduced risk of premature rupture of membranes, while genus *Collinsella*, genus *Intestinibacter* and genus *Turicibacter* increased the risk for this condition. In addition, Chen et al. examined the potential causal connections between gut microbiota and rheumatic valve disease using a Mendelian randomization framework. They found *Lentisphaerae*, *Alphaproteobacteria*, and *Streptococcaceae* as having significant protective effects against rheumatic valve disease, whereas *Eubacterium eligens* and *Odoribacter* were identified as potential risk factors. These findings were mediated by specific immune cell traits and biomarkers. Moreover, Zhong et al. provided genetic evidence linking gut microbiota to colorectal cancer. Specifically, *Bifidobacterium kashiwanohense, GCA-900066755* sp*900066755, Geminocystis*, and *Saccharofermentanaceae* exhibited robust causal effects for this disease, mediated by specific circulating immune cells. Furthermore, Tian et al. reported an association between multiple bacterial genera and epigenetic clocks, which was mediated by inflammatory cytokines. Their study suggests a genetic relationship between gut microbiota and aging, providing new avenues for aging-related research and the development of new treatment modalities.

The role of the intestinal microbiota in regulating the immune system has attracted attention in inflammatory diseases. In this context, Qi et al. reviewed the complex interactions between gut microbiota homeostasis and immune regulation in rheumatoid arthritis pathogenesis. They highlighted that the imbalance in the composition and function of the gut microbiota (dysbiosis) may increase gut permeability, release pro-inflammatory molecules, and impair regulatory T cell function. These disruptions collectively contribute to immune dysregulation, ultimately driving the onset and progression of rheumatoid arthritis.

The microbiota–gut–brain axis, a bidirectional connection between the gut microbiota and the brain through the relevant pathways of the gut–brain axis, may play a role in the pathogenesis of neurological disorders, including epilepsy. In this regard, You et al. investigated relationships between the gut microbiota, the hypothalamus-pituitary-adrenal axis hormones, and the inflammatory cytokines in children with infantile spasms. The authors found that dysbiosis may be involved in the pathogenesis of infantile spasms and is related to the response to adrenocorticotropic hormone. Specifically, *Lachnospiraceae* and *Lachnospiracea_incertae_sedis* appear to be involved in disease onset, while *Sutterellaceae* may play a role in children’s improved health.

Overall, the articles published on this Research Topic provide valuable insights into the relationship between microbiota and epigenetics in relation to host health. This knowledge enhances our understanding of inflammatory and neurological diseases and aids in identifying therapeutic targets that might be exploited through innovative intervention strategies. Future research in this field includes the analysis of different epigenetic mechanisms affected by alterations in the gut microbiota in highly prevalent metabolic diseases, such as obesity, type 2 diabetes, and fatty liver disease. Additionally, integrating other omics technologies, such as metabolomics, will strengthen the analysis of epigenetic regulation by metabolites from the gut microbiome in health and diseases. The role of environmental factors that modulate the microbiota and epigenome such as diet, exercise, sleep patterns, and emotions, should also be considered in this holistic vision.
